# Supplementary material for: A conserved tooth resorption mechanism in modern and fossil snakes
Source: Nat Commun. 2023 Feb 10;14:742. doi: 10.1038/s41467-023-36422-2 (PMC9918488; doi:10.1038/s41467-023-36422-2)
Supplement: Supplementary file 1 — Supplementary Information [file 41467_2023_36422_MOESM1_ESM.pdf]

A conserved tooth resorption mechanism in modern and fossil snakes

SUPPLEMENTARY INFORMATION

LeBlanc, A. R. H., Palci, A., Anthwal, N., A., Tucker, A. S., Araújo, R., Pereira, M. F. C., and Caldwell, M.W.

[1]Department of Biological Sciences, University of Alberta, Edmonton, Alberta, Canada
[2]Centre for Oral, Clinical & Translational Sciences, King's College London, United Kingdom
[3]School of Biological Sciences, University of Adelaide, Adelaide, South Australia, Australia
[4]South Australian Museum, Adelaide, South Australia, Australia
[5]Centre for Craniofacial & Regenerative Biology, King's College London, United Kingdom
[6]Instituto de Plasmas e Fusão Nuclear, Instituto Superior Técnico, Universidade de Lisboa, Lisbon, Portugal
[7]CERENA, Instituto Superior Técnico, Universidade de Lisboa, Lisbon, Portugal

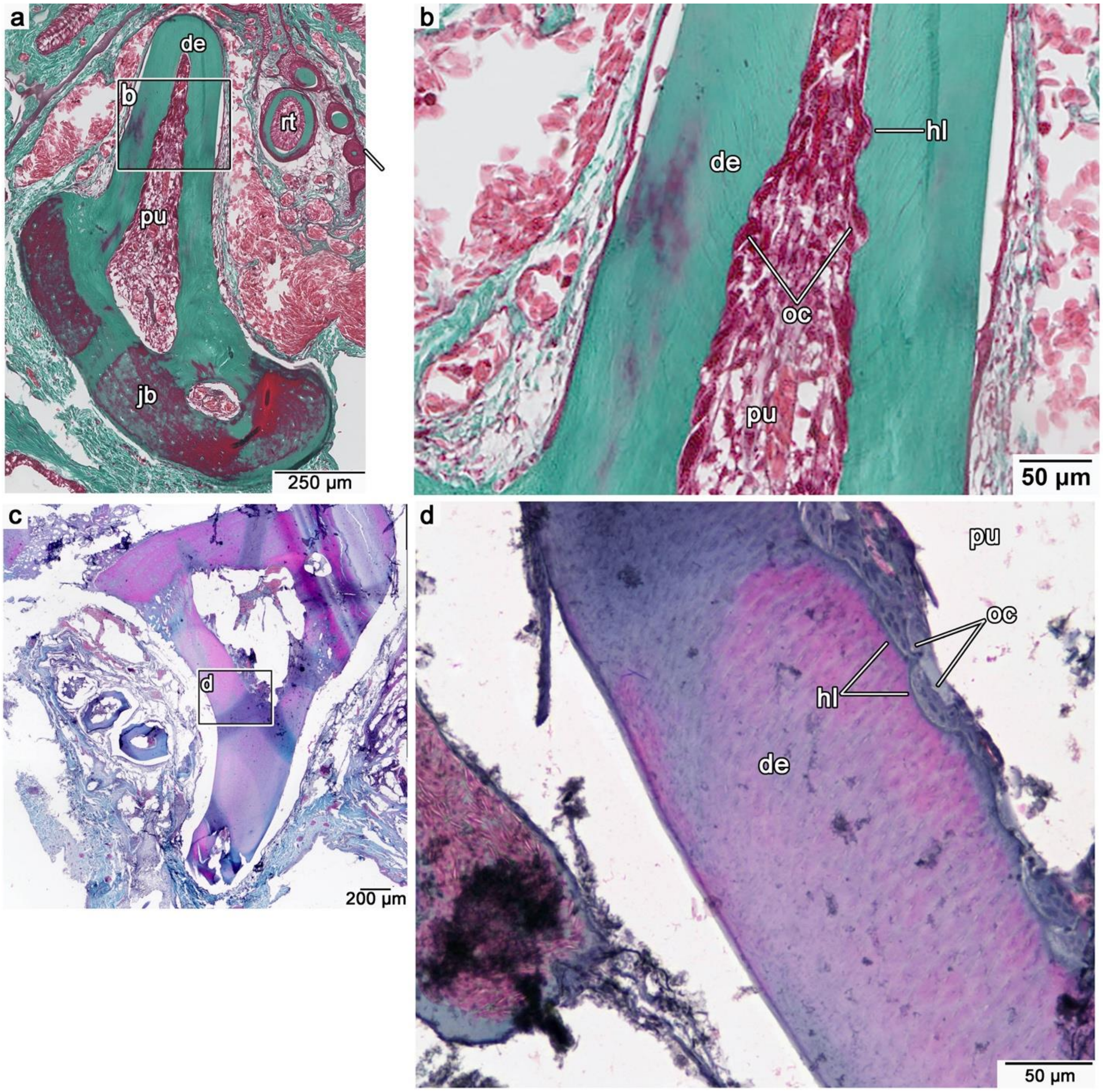

Supplementary Figure 1**. Internal tooth resorption in histological and digital sections of *Oxyuranus scutellatus* and *Acrochordus javanicus*. a** Transverse section through a dentary tooth of a taipan (*Oxyuranus scutellatus*) showing initial stages of tooth resorption. **b** Closeup of the pulp of the tooth in (A), showing internal odontoclastic resorption. **c** Section through a pterygoid tooth of *Acrochordus javanicus* (H&E). **d** Closeup of the internal wall of the pterygoid tooth in c showing early stages of tooth resorption by odontoclasts found within the pulp. Abbreviations: de, dentine; hl, Howship's lacunae; oc, odontoclasts; pu, pulp. Black arrows indicate centrifugal resorption of the dentine.

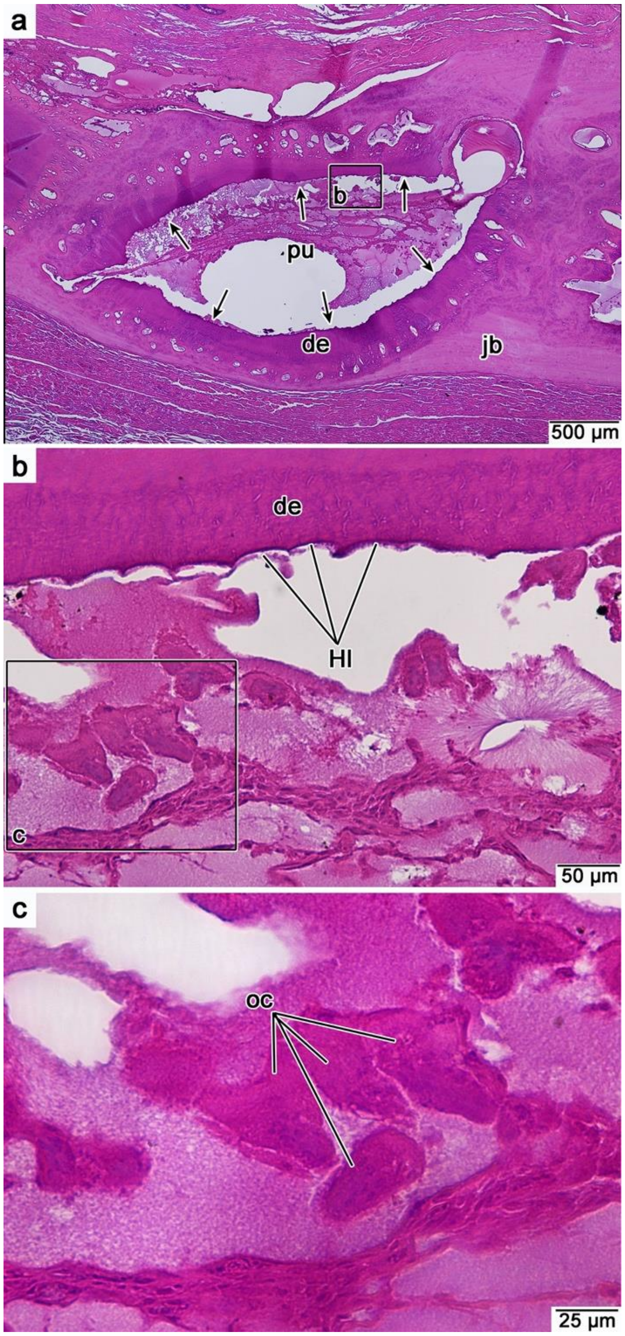

Supplementary Figure 2. **Histology of snake-type tooth replacement in horizontal section. a** Early stages of internal (pulpal) resorption in a maxillary tooth base of *Boa constrictor* (hematoxylin and eosin staining). **b** Closeup of the lingual dentine surface in a showing Howship's lacunae and associated odontoclasts along the inner dentine wall. **c** Closeup of multinucleated, pulpal odontoclasts in b. Note that the odontoclasts have separated from the dentine surface, most likely due to the poor preservation of the soft tissues in this freezer-stored specimen. Abbreviations: de, dentine; hl, Howship's lacunae; jb, bone of the jaw; oc, odontoclasts; pu, pulp. Arrows indicate directions of resorption based on the occurrences of Howship's lacunae.

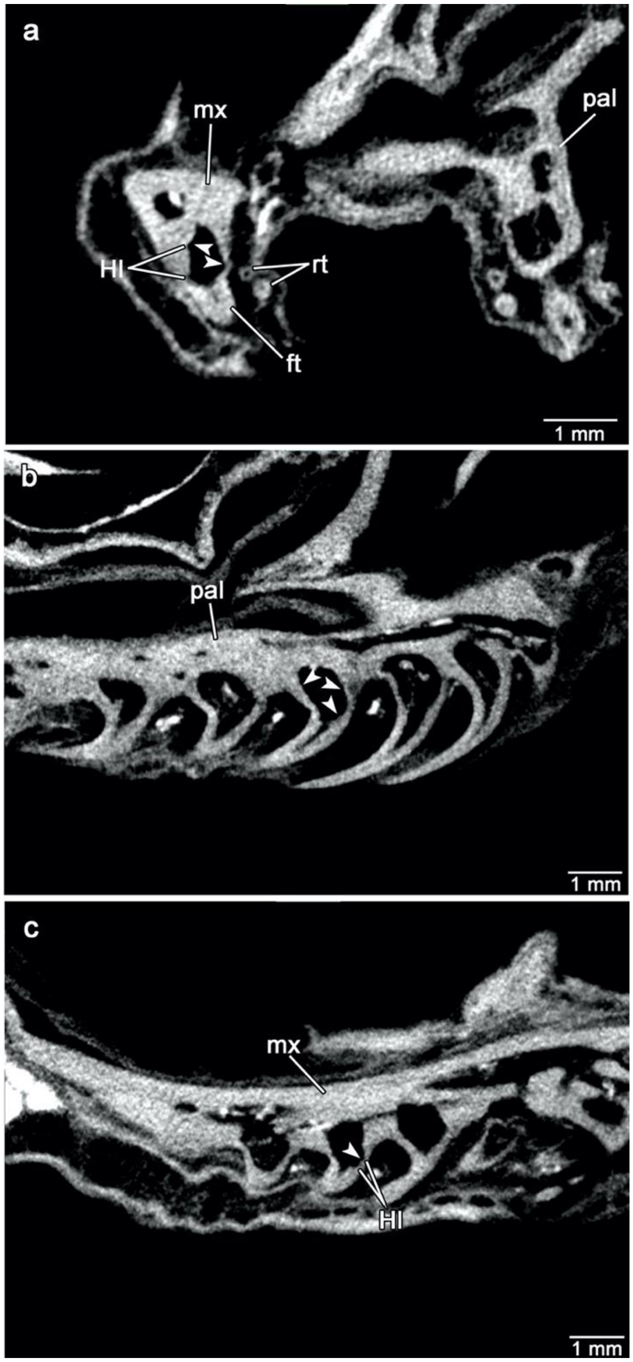

Supplementary Figure 3. **Internal tooth resorption in the maxillary and palatine teeth of the colubrid snake *Boiga dendrophila* as detected by µCT scanning. a** Digital coronal section of the skull showing internal resorption and Howship's lacunae in a maxillary tooth. **b** Digital parasagittal section through the palatine of *Boiga dendrophila* showing internal resorption in a palatal tooth. **c** Digital parasagittal section through the maxilla, showing internal resorption and distinct Howship's lacunae in a maxillary tooth. Arrowheads indicate directions of resorption. Abbreviations: ft, functional tooth; Hl, Howship's lacunae; mx, maxilla; pal, palatine; rt, replacement teeth.

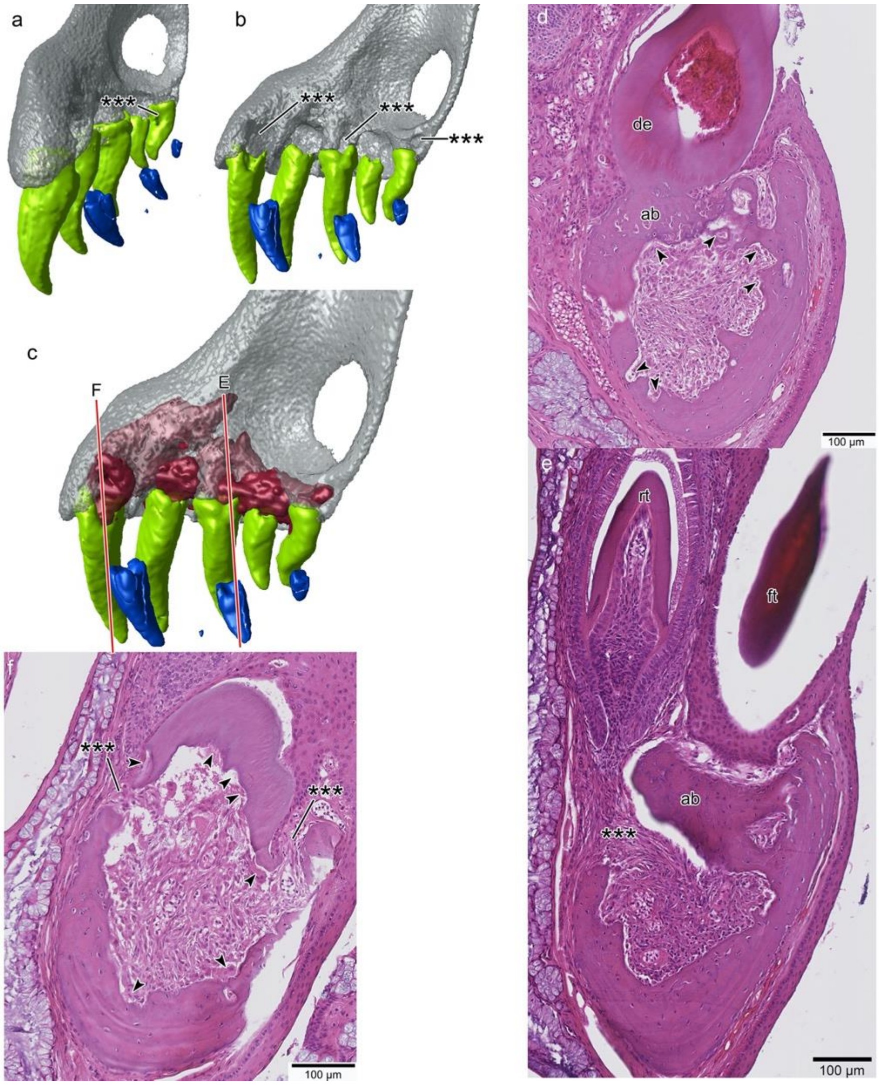

Supplementary Figure 4. **Internal tooth resorption in the scolecophidian snake, *Anilios bicolor*. a** μCT digital model of the maxilla of SAMA R1065 showing collateral tooth resorption (astersisks) to the smallest tooth, presumably caused by the resorption and shedding of the neighbouring tooth position, now occupied by a replacement tooth in the process of attaching to the maxilla. **b** Lingual view of the maxilla showing positions of three external pits (asterisks), progressing from the maxilla (right-most tooth position) and eventually on to the tooth base (left-most tooth). **c** Lingual view of the maxilla highlighting the internal resorption within the maxilla (segmented in red), which is continuous with the external pits visible in b. **d** H&E stained section of the other maxilla (not figured) showing the extent of internal resorption within the maxilla, below a tooth. **e** Section through the middle tooth in c, showing the externally visible pit (asterisks) within the maxilla, which seems to be more closely related to the internal maxillary resorption than with the replacement tooth farther away. **f** Section through left-most tooth in c showing the initial breach of the pulp by the replacement tooth (left asterisks), but with a completely transformed pulp, with extensive resorption. The resorption within the pulp has proceded so far that the labial margin of the tooth base has also detached from the maxilla (right astersisks). Abbreviations: ab, alveolar bone; de, dentine; ft, functional tooth; rt, replacement tooth

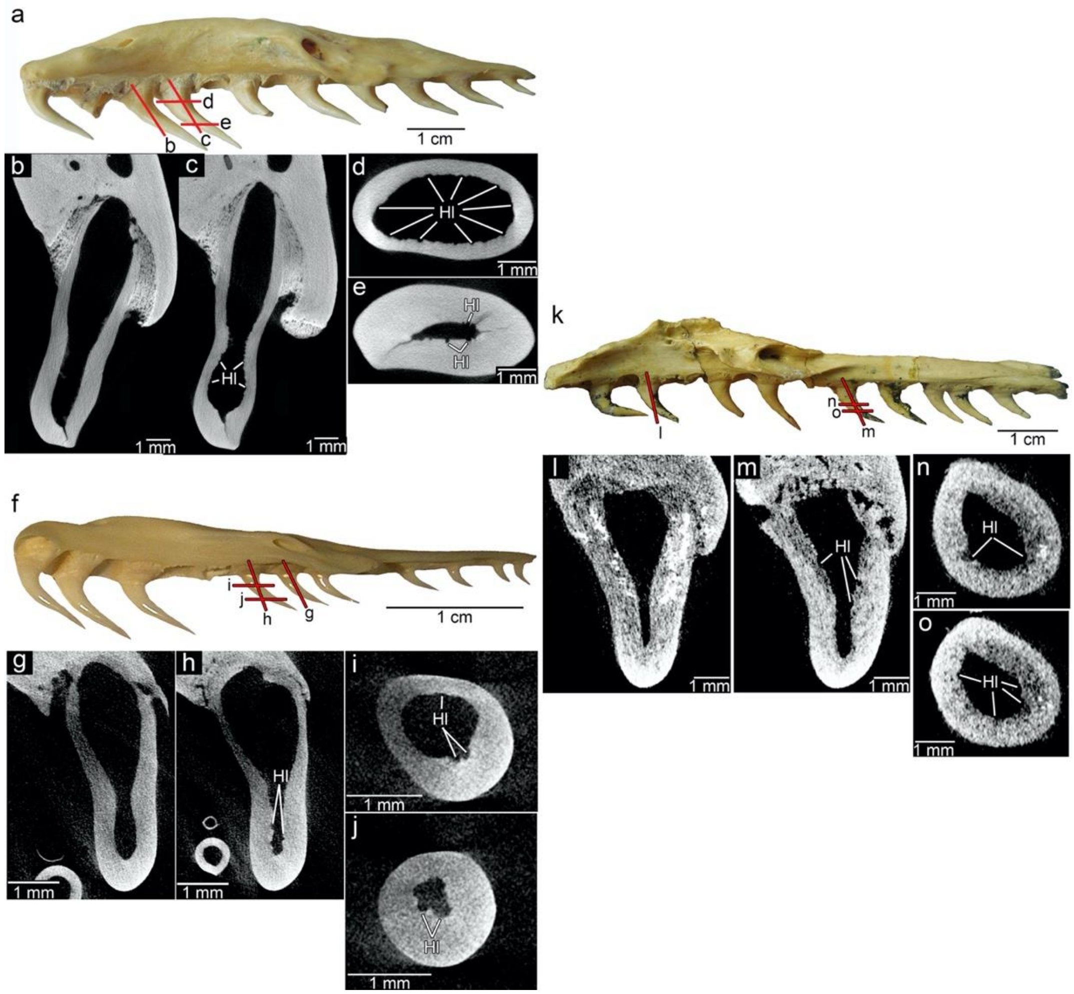

Supplementary Figure 5. **Evidence for internal resorption from µCT scans of modern and fossil snakes. a** Isolated maxilla of a *Malayopython reticulatus* (SAMA R27307) showing positions of CT slices in subsequent panels. **b** Transverse section through a maxillary tooth of *M. reticulatus* that has not undergone internal resorption. **c** Transverse section through a maxillary tooth crown of *M. reticulatus* undergoing internal resorption. **d** Horizontal section through the same maxillary tooth in c taken close to the tooth base, showing internal resorption. **e** Same as in d, but where the cutaway plane is higher through the crown. **f** Isolated maxilla of a *Boa constrictor* [Alexander Koenig Research Museum, Bonn, Germany (ZFMK) 21662] showing approximate positions of CT slices in subsequent panels. **g** Coronal section of a newly attached maxillary tooth showing smooth, unresorbed internal surfaces of the dentine. **h** Coronal section showing evidence of advanced internal resorption, even up into the pulp of the crown. **i** Transverse section through the base of a tooth undergoing internal resorption. **J** Transverse section through the same tooth shown in (h, i), taken closer to the tooth crown tip. **k** Maxilla of the fossil snake *Yurlunggur* sp. (QMF45391) showing positions of CT slices in subsequent panels. **l** Transverse section of an unresorbed maxillary tooth of *Yurlunggur*. **m** Transverse section through a partially resorbed maxillary tooth. **n** Horizontal section through a partially resorbed maxillary tooth near its base. **o** Horizontal section through the same tooth taken at a slightly different level closer to the crown. Abbreviations: Hl, Howship's lacunae.

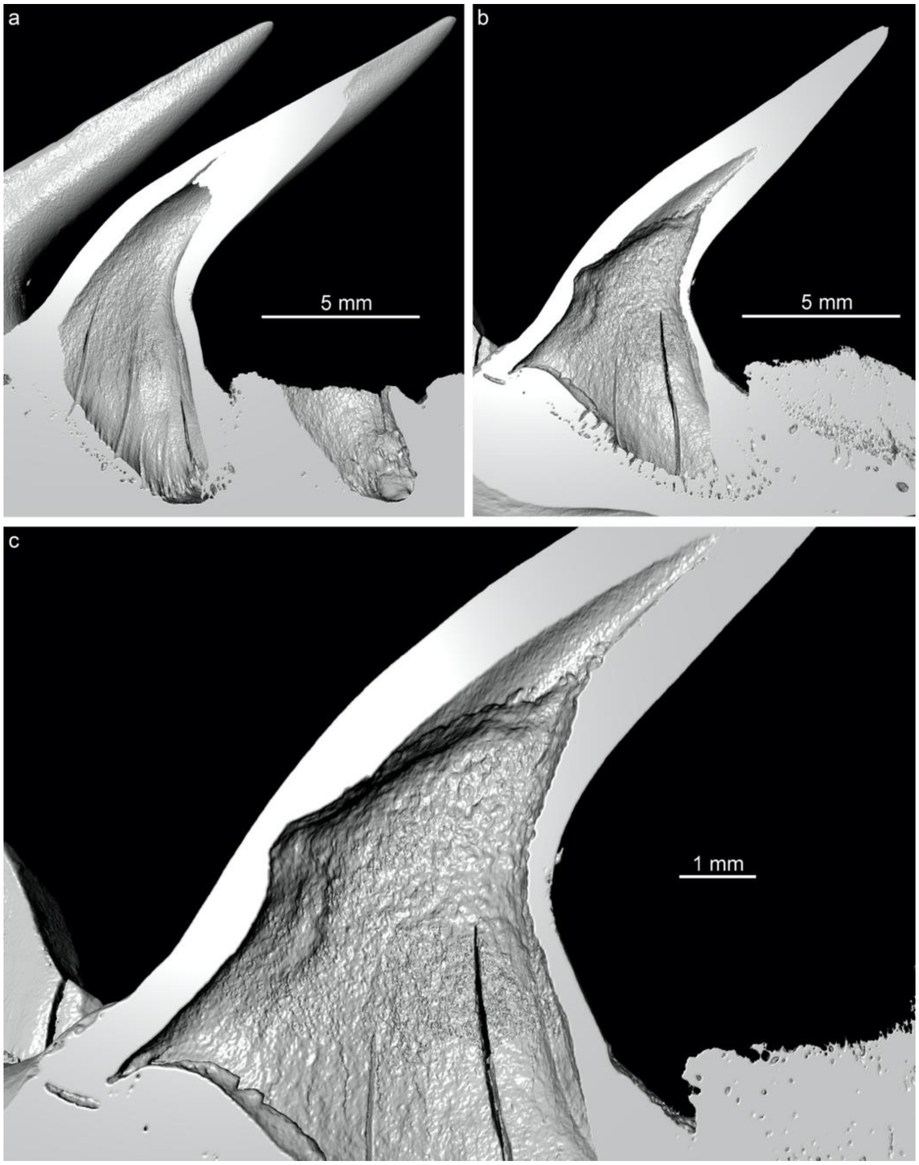

Supplementary Figure 6. **Three-dimensional CT reconstruction of unresorbed and internally resorbed teeth in a skeletonized maxilla of *Malayopython reticulatus* (SAMA R27307).** Images are fipped. **a** Digital sagittal section through an unresorbed tooth. **b** Sagittal section showing partial internal resorption of a tooth undergoing replacement. **c** Closeup of scalloped internal dentine wall of the tooth in b. Dark lines are desiccation cracks.

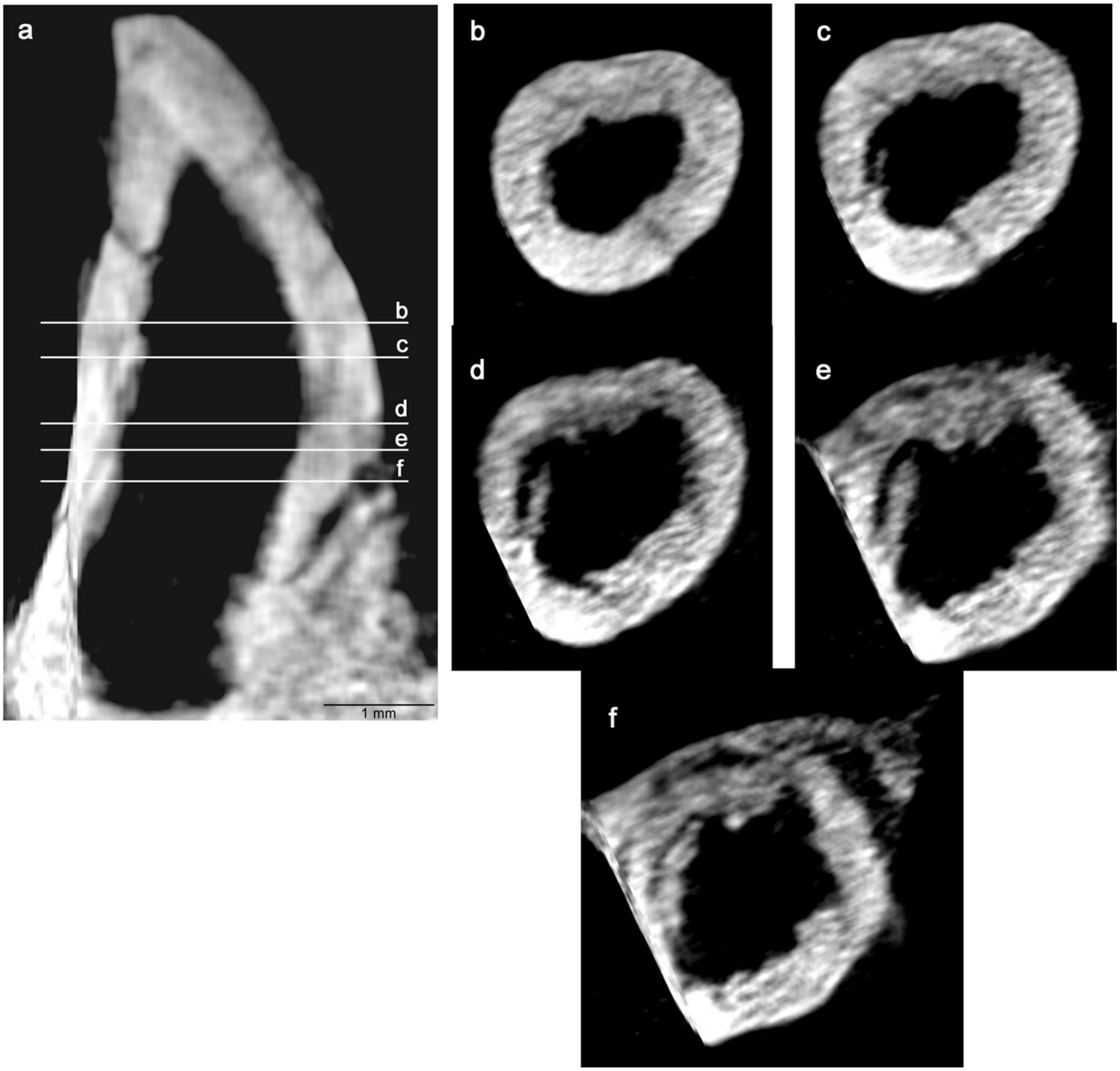

Supplementary Figure 7. **μCT slices through the fifth dentary tooth position in the *Portugalophis lignites* dentary (MG-LNEG 28094) showing the scalloped internal dentine wall.** The first four transverse sections are taken above the alveolar margin of the dentary, indicating that the internal scalloping is likely caused by odontoclast activity and not folded dentine, which occurs at the bases of the teeth in other snakes[1]. **a** Sagittal slice through the tooth showing positions of horizontal slices in b-f. **b** Horizontal section taken closest to the crown top showing large Howship's lacunae near the top of the pulp cavity in the image. **c** Slightly lower horizontal section showing increasing number of Howship's lacunae along the inner wall of the tooth. **d** More apical horizontal section showing more extensive Howship's lacunae. **e** Horizontal section slightly above the alveolar margin showing more abundant Howship's lacunae. **f** Horizontal section at level of alveolar margin showing extensive internal resorption. In all images, anterior is to the right.

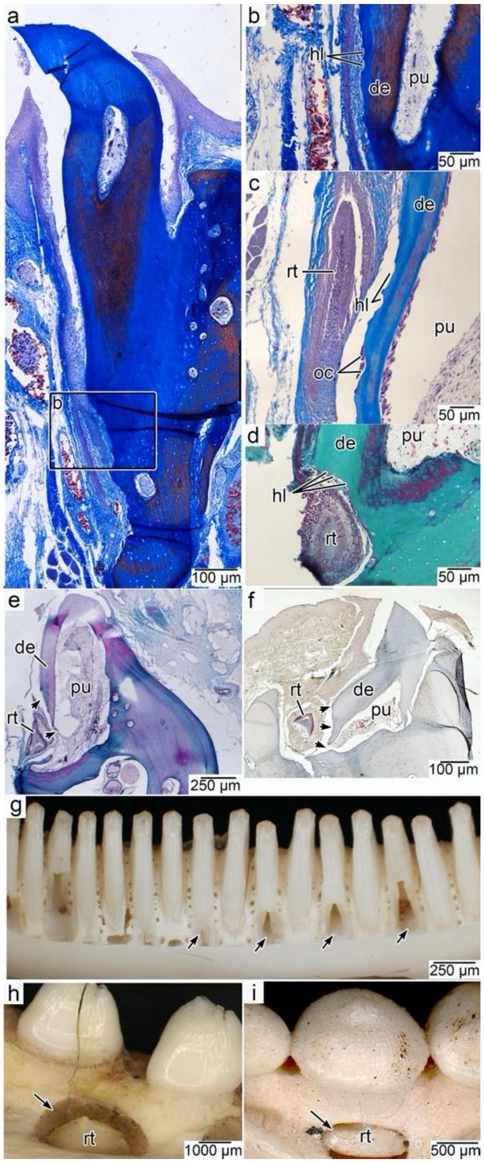

Supplementary Figure 8. **Early stages of resorption in the iguanid-type tooth replacement cycle in various lizard species. a** Transverse section through a dentary tooth of the iguanid *Sauromalus ater* (Masson's trichrome stain). **b** Closeup of the initial phase of tooth resorption in a. **c** Closeup of the initial phase of tooth resorption in a coronal section of an *Iguana iguana* dentary tooth (Masson's trichrome stain). **d** Closeup of the initial phase of tooth resorption in a coronal section of a dentary tooth in the teiid *Aspidoscelis exsanguis* (Gomori's trichrome stain). **e** Transverse section through a tooth of *Scincus scincus* showing the development of a resorption pit that has breached the pulp (H&E stain). Black arrows indicate directions of resorption. **f** Transverse section through a tooth of *Cordylus cordylus* showing the development of a resorption pit in advance of the approaching replacement tooth (H&E stain). Black arrows indicate directions of resorption. **g** Lingual view of a dentary tooth row of *C. cordylus* showing increasing sizes of resorption pits (black arrows). **h** Early-stage resorption pit (black arrow) and associated replacement tooth in a specimen of *Tupinambis teguixin*. **i** Resorption pit (black arrow) and associated replacement tooth in a *Dracaena guianensis* dentary. Abbreviations: de, dentine; hl, Howship's lacunae; oc, odontoclast; pu, pulp; rt, replacement tooth.

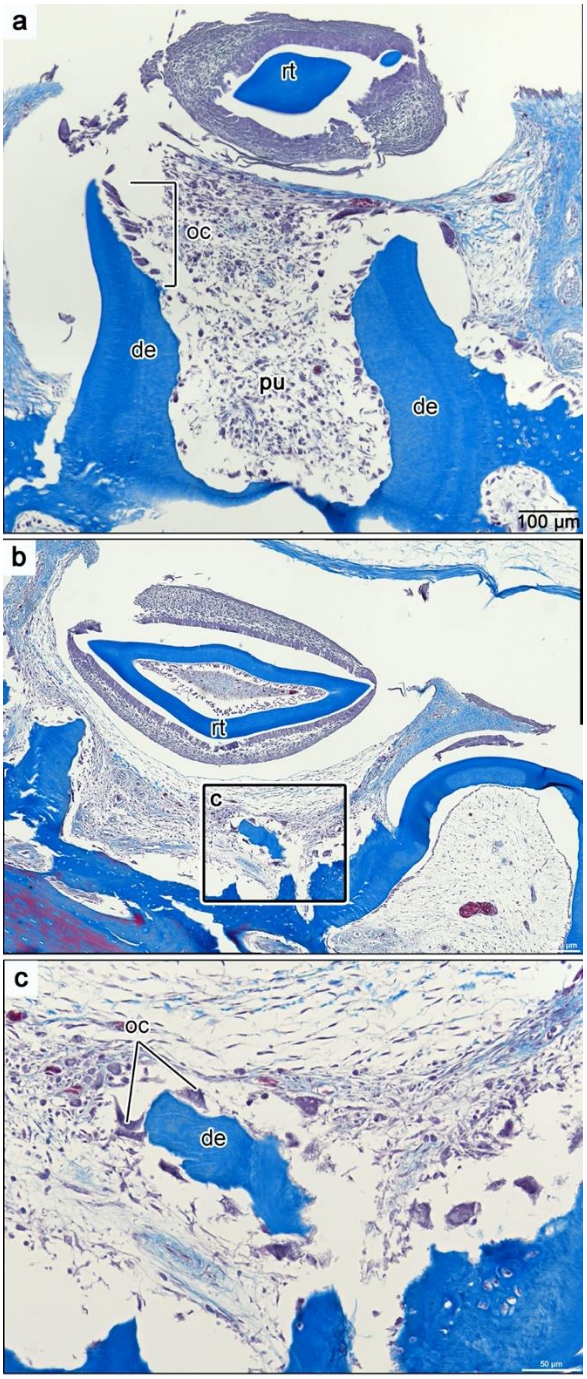

Supplementary Figure 9. **Advanced stages of tooth resorption in the iguanid-type tooth replacement cycle. a** Resorption of the internal walls of the functional tooth and expansion of the resorption pit in an *Iguana iguana* dentary tooth (Masson's trichrome staining). The odontoclasts at this stage begin resorbing the internal dentine walls. **b** Complete resorption of the functional tooth base and the eruption of the replacement tooth in an *I. iguana* tooth (Masson's trichrome stain). **c** Closeup of a region in b showing a small island of dentine from the functional tooth being resorbed by a cluster of odontoclasts. Abbreviations: de, dentine; oc, odontoclasts; pu, pulp; rt, replacement tooth.

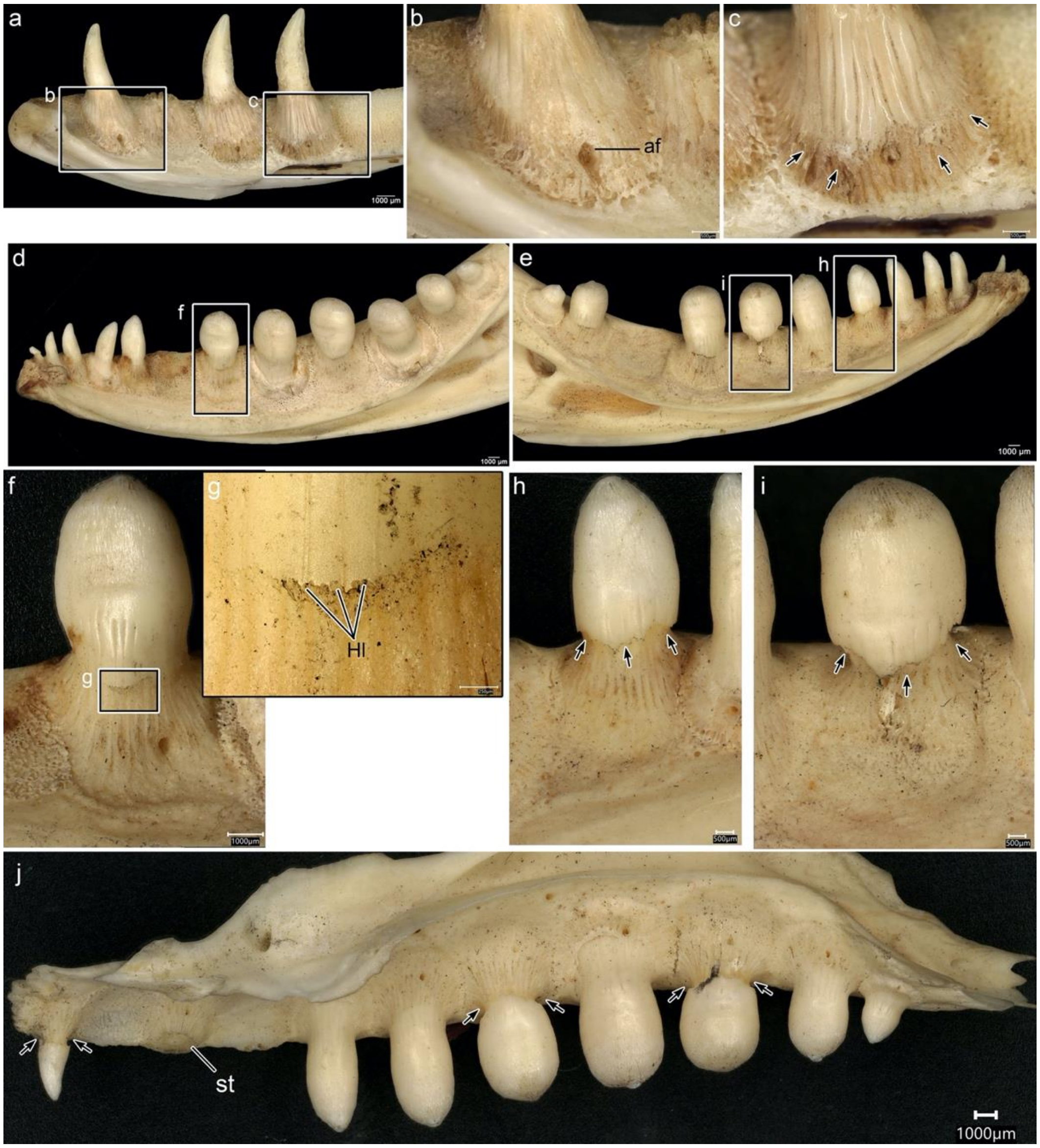

Supplementary Figure 10. **Osteological signs of the tooth replacement cycle in *Varanus*. (A) lingual view of a right dentary of *Varanus* sp. (MoLS X265). b** Closeup of a dentary tooth lacking signs of resorption. **c** Closeup of a dentary tooth showing early signs of external resorption along the tooth base (black arrows). **d** Lingual view of a right dentary of *Varanus niloticus* (MoLS X266). **e** Lingual view of the left dentary of the same *V. niloticus* specimen. **f** closeup of a mid-dentary tooth showing early stages of external resorption around the base of the tooth. **g** closeup image of tooth resorption in (F) showing the presence of Howship's lacunae. **(H)** closeup of left dentary tooth showing more advanced stage of tooth resorption (black arrows), which has eroded away the base of the tooth from all directions. **(I)** closeup of left dentary tooth showing further advancement of tooth resorption (black arrows), where the tooth base is nearly complete eroded away from all sides. **(J)** lingual view of right maxilla of *V. niloticus* showing signs of external tooth resorption and tooth shedding. Abbreviations: af, alveolar foramen; Hl, Howship's lacunae; st, shed tooth.

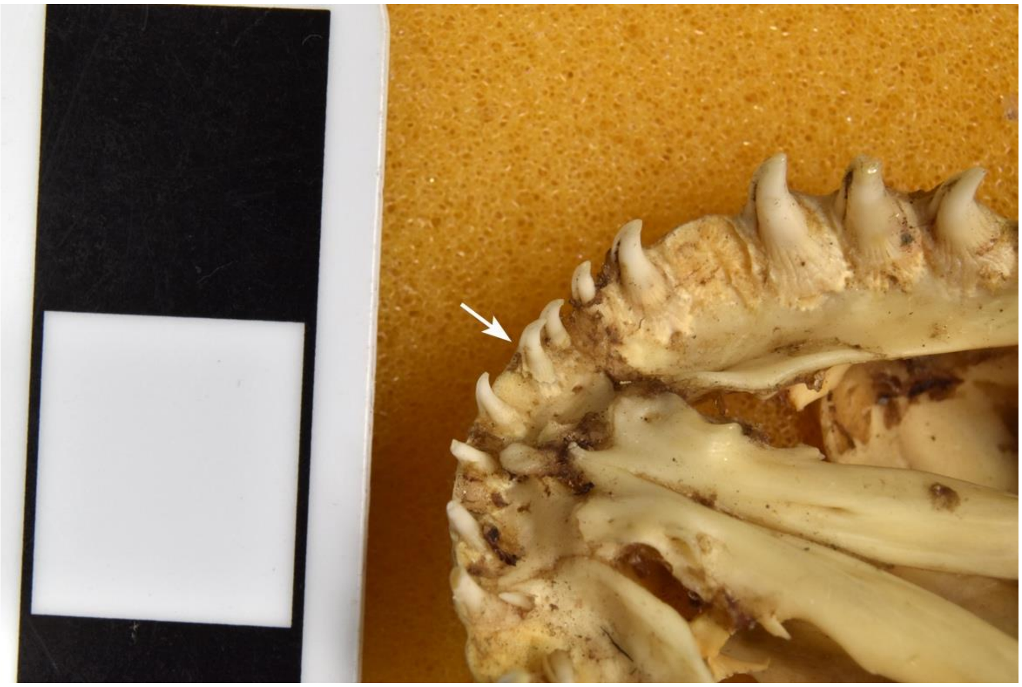

Supplementary Figure 11. **Ventral view of the skull of *Heloderma horridum* ("II. 1a", BMNHUK collections) showing a premaxillary tooth (white arrow) with identical undercutting external resorption as that described here for *Varanus* (see Supplementary Fig. 10).**

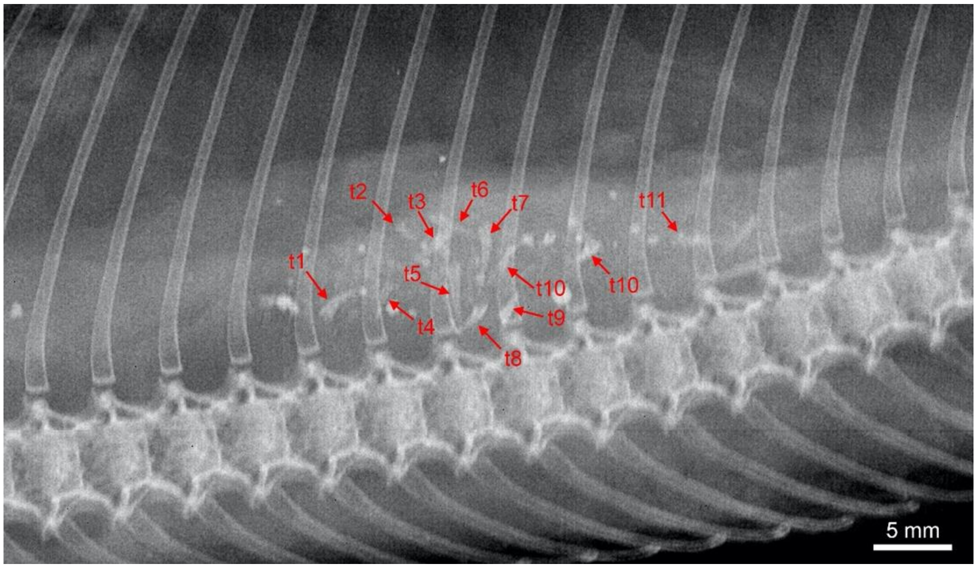

Supplementary Figure 12. **Radiograph of the precloacal region of an ethanol-preserved specimen of *Python curtus* (ZFMK 76303).** At least eleven teeth (t1-t11) can be identified in what was the last meal of this snake, providing an example of the large number of teeth that can be shed by snakes while feeding (ZFMK = Zoological Research Museum Alexander Koenig, Bonn, Germany).

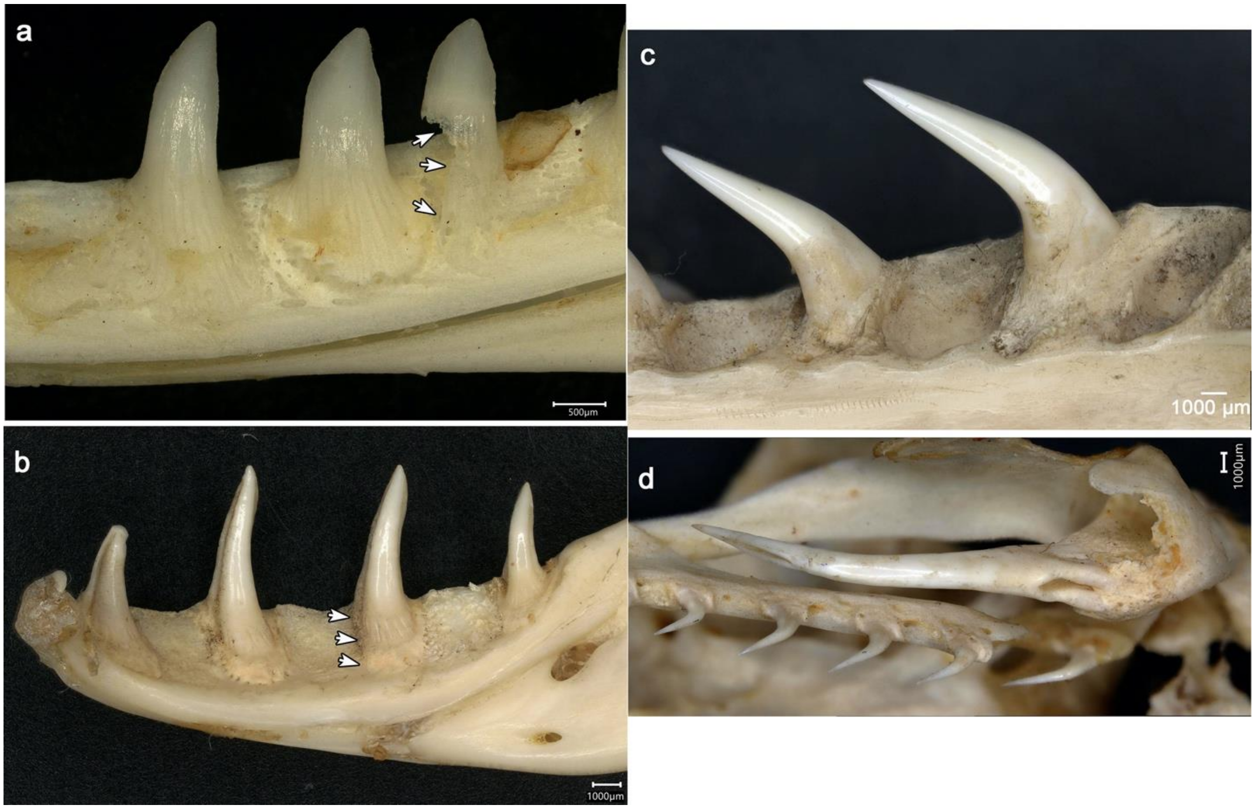

Supplementary Figure 13. **Evidence for collateral resorption to neighbouring tooth bases in varanoids and a lack of collateral resorption in snakes. a** Lingual view of a dentary of *Varanus bengalensis* (MoLS X38) showing extensive collateral resorption (white arrows) caused by the growth and replacement of the tooth in the middle. **b** Lingual view of a dentary of *Heloderma horridum* (MoLS X185) showing collateral resorption (white arrows) to the tooth base of a neighbouring tooth following the shedding of an adjacent tooth due to external resorption. **c** Lingual view of dentary teeth in *Python* sp. (MoLS X198) showing lack of collateral resorption caused by shedding of neighbouring teeth. Instead, interdental ridges have accumulated between closely packed, adjacent teeth. **d** Ventral view of the maxillary venom fang of *Bitis* sp. (MoLS X201) showing the lack of collateral resorption from the shedding of the adjacent venom fang. Venom fangs are often closely packed together, but show very little evidence of collateral resorption.

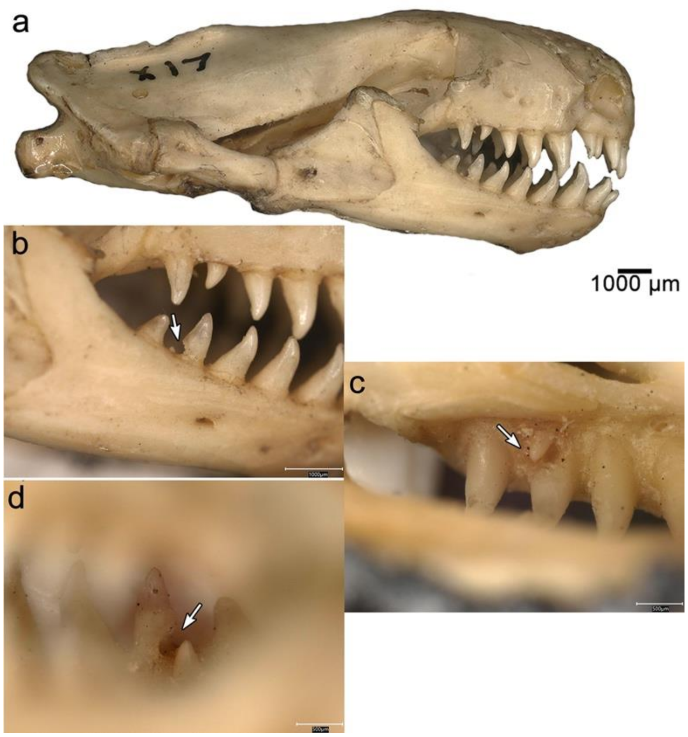

Supplementary Figure 14. **External, iguanid-type tooth replacement in *Amphisbaena* sp. (MoLS X17). a** Skull in right lateral view. **b** Lateral view of a dentary tooth showing a large resorption pit (white arrow). **c** Lingual view of a maxillary tooth with a small replacement tooth and associated resorption pit. **d** Lingual view of dentary tooth in b showing the replacement tooth and resorption pit.

Supplementary References

1. Palci, A. *et al.* Plicidentine and the repeated origins of snake venom fangs. *Proc. R. Soc. B Biol. Sci.* **288**, 20211391 (2021).
