# Supplementary material for: A conserved tooth resorption mechanism in modern and fossil snakes
Source: Nat Commun. 2023 Feb 10;14:742. doi: 10.1038/s41467-023-36422-2 (PMC9918488; doi:10.1038/s41467-023-36422-2)
Supplement: Supplementary file 3 — Description of Additional Supplementary Files [file 41467_2023_36422_MOESM3_ESM.pdf]

Description of Additional Supplementary Material

Supplementary Data 1. **Table of specimens examined in this study.**

Supplementary Movie 1. **Virtual frontal sections through a maxillary tooth of *Boa constrictor* lacking internal tooth resorption.**

Supplementary Movie 2. **Virtual frontal sections through a maxillary tooth of *Boa constrictor* exhibiting internal tooth resorption and Howship's lacunae.**

Supplementary Movie 3. **Virtual horizontal sections through a maxillary tooth of *Boa constrictor* exhibiting internal tooth resorption and Howship's lacunae.**

Supplementary Movie 4. **Virtual frontal sections through a maxillary tooth of *Malayopython reticulatus* lacking internal tooth resorption.**

Supplementary Movie 5. **Virtual frontal sections through a maxillary tooth of *Malayopython reticulatus* exhibiting internal tooth resorption and Howship's lacunae.**

Supplementary Movie 6. **Virtual frontal sections through a maxillary tooth of *Yurlunggur* lacking internal tooth resorption.**

Supplementary Movie 7. **Virtual frontal sections through a maxillary tooth of *Yurlunggur* exhibiting internal tooth resorption and Howship's lacunae.**

Supplementary Movie 8. **Virtual horizontal sections through a dentary tooth of *Portugalophis lignites* exhibiting internal tooth resorption and Howship's lacunae.**
