# Supplementary material for: A conserved tooth resorption mechanism in modern and fossil snakes
Source: Nat Commun. 2023 Feb 10;14:742. doi: 10.1038/s41467-023-36422-2 (PMC9918488; doi:10.1038/s41467-023-36422-2)
Supplement: Supplementary file 13 — Reporting Summary [file 41467_2023_36422_MOESM13_ESM.pdf]

nature portfolio

Corresponding author(s): Aaron R. H. LeBlanc

Last updated by author(s): 2023/01/26

Reporting Summary

Nature Portfolio wishes to improve the reproducibility of the work that we publish. This form provides structure for consistency and transparency in reporting. For further information on Nature Portfolio policies, see our Editorial Policies and the Editorial Policy Checklist.

Statistics

For all statistical analyses, confirm that the following items are present in the figure legend, table legend, main text, or Methods section.

n/a	Confirmed	
☐	☒	The exact sample size (*n*) for each experimental group/condition, given as a discrete number and unit of measurement
☒	☐	A statement on whether measurements were taken from distinct samples or whether the same sample was measured repeatedly
☒	☐	The statistical test(s) used AND whether they are one- or two-sided<br>*Only common tests should be described solely by name; describe more complex techniques in the Methods section.*
☒	☐	A description of all covariates tested
☒	☐	A description of any assumptions or corrections, such as tests of normality and adjustment for multiple comparisons
☒	☐	A full description of the statistical parameters including central tendency (e.g. means) or other basic estimates (e.g. regression coefficient) AND variation (e.g. standard deviation) or associated estimates of uncertainty (e.g. confidence intervals)
☒	☐	For null hypothesis testing, the test statistic (e.g. *F*, *t*, *r*) with confidence intervals, effect sizes, degrees of freedom and *P* value noted<br>*Give P values as exact values whenever suitable.*
☒	☐	For Bayesian analysis, information on the choice of priors and Markov chain Monte Carlo settings
☒	☐	For hierarchical and complex designs, identification of the appropriate level for tests and full reporting of outcomes
☒	☐	Estimates of effect sizes (e.g. Cohen's *d*, Pearson's *r*), indicating how they were calculated

Our web collection on statistics for biologists contains articles on many of the points above.

Software and code

Policy information about availability of computer code

Data collection	No software was used for data collection.
Data analysis	Avizo Lite v.9.0 (Thermo Scientific); Dragonfly v.4.1 (Object Research Systems Inc.).

For manuscripts utilizing custom algorithms or software that are central to the research but not yet described in published literature, software must be made available to editors and reviewers. We strongly encourage code deposition in a community repository (e.g. GitHub). See the Nature Portfolio guidelines for submitting code & software for further information.

Data

Policy information about availability of data

All manuscripts must include a data availability statement. This statement should provide the following information, where applicable:

- Accession codes, unique identifiers, or web links for publicly available datasets
- A description of any restrictions on data availability
- For clinical datasets or third party data, please ensure that the statement adheres to our policy

All of the thin section images used for this study are either in the main text of the manuscript or in the Supplementary Information. We have included videos of the reconstructed µCT slices through pairs of resorbing and non-resorbing teeth of Boa constrictor, Malayopython reticulatus, Yurlunggur sp., and Portugalophis lignites (Supplementary Videos 1–8). The µCT datasets for Boa constrictor and Yurlunggur are available on Morphosource (Project: Teeth of Serpentes, https://www.morphosource.org/projects/000358042?locale=en). The µCT datasets for Malayopython reticulatus, Boiga dendrophila, Crotalus atrox, Anilios bicolor, and Portugalophis lignites used in this study are available on Morphosource (Project Modern and fossil snake tooth replacement, https://www.morphosource.org/

projects/000395615?locale=en).

Human research participants

Policy information about studies involving human research participants and Sex and Gender in Research.

Reporting on sex and gender	N/A
Population characteristics	N/A
Recruitment	N/A
Ethics oversight	N/A

Note that full information on the approval of the study protocol must also be provided in the manuscript.

Field-specific reporting

Please select the one below that is the best fit for your research. If you are not sure, read the appropriate sections before making your selection.

☐ Life sciences ☐ Behavioural & social sciences ☒ Ecological, evolutionary & environmental sciences

For a reference copy of the document with all sections, see nature.com/documents/nr-reporting-summary-flat.pdf

Ecological, evolutionary & environmental sciences study design

All studies must disclose on these points even when the disclosure is negative.

Study description	This study involves non-destructive micro-CT imaging and histological sectioning of samples of extant snakes and lizards, and CT imaging of two fossil snakes, one from Australia and the other from Portugal. MicoCT scanning occurred in four different labs using different scanning machines (University of Alberta, University of Adelaide, University of Lisbon, and King's College London, histological preparation was done in three institutions (University of Alberta, University of Adelaide, and King's College London). Tartrate-Resistant Acid Phosphatase (TRAP) staining was conducted on a single fluid-preserved corn snake head. Haematoxylin and Eosin as well as Masson's Trichrome staining were used for the remaining samples.
Research sample	Preserved collections of extant snakes and lizards from collections at King's College London, University of Alberta, and the South Australian Museum were histologically sampled for this study, based on their availability to the researchers, the phylogenetic positions of each species, and permissions for destructive sampling or CT scanning. Some of the micro-CT scans and other extant histological samples had already been collected in two previous studies (Palci et al., 2020, Proceedings of the Royal Society B 288: 20211391; LeBlanc et al. 2021, Journal of Anatomy 238: 1156-1178). See Supplementary Data 1 for detailed sample provenance.
Sampling strategy	Samples were chosen based on specimen availability at the respective institutions (relative abundance of species in museum collections, appropriateness for destructive histological sampling). Samples of extant snakes were also chosen based on their phylogenetic positions within Pan-Serpentes. Specimens belonging to major clades Scolecophidia, Constrictores, Acrochordidae, Viperidae, Colubridae, and Elapidae are interpreted as representatives for their respective species and higher level clades. Sex, age, and population were not considered for any sample. Fossils of Yurlunggur and Portugalophis were chosen based on the presence of multiple teeth along isolated jaws that could be non-destructively sampled via micro-CT scanning.
Data collection	Histological data were collected by the authors (A. LeBlanc, A. Palci, N, Anthwal). Snake heads were decalcified and sectioned using rotary microtomes. Sections were mounted to glass slides and stained using various staining techniques (see Methods). Histology images were taken under microscopes using mounted digital cameras with imaging software. CT scanning was conducted by A. Palci, N. Anthwal, M.F.C. Pereira, and R. Araujo using a Skyscan 1176, Skyscan 1076, Skyscan 1172, and Scanco MicroCT 50.
Timing and spatial scale	Data were collected from 2018-2022 and depended on sample availability. Histological sectioning and/or scanning of Hydrophis cyanocinctus, Boa constictor, Yurlunggur, and Iguana iguana were made for previous studies in 2018. Sectioning of Boa constrictor and Varanus sp. occurred between 2018 and 2019. Scanning of Portugalophis lignites occurred in 2021. Scans of Anilios bicolor were made in 2018 for a previous study. Thin sectioning of A. bicolor occurred in 2022. All other non-snake lizard sections were made for an MSc thesis in 2002-2003 and were imaged for this study between 2018-2020. Images of museum specimens were taken over several collection visits between 2020 and 2022.
Data exclusions	None.
Reproducibility	Histological sections are stored in their respective institutions (University of Adelaide, University of Alberta, King's College London) and are available for study. Fossils of Yurlunggur and Portugalophis are accessioned in publicly accessible institutions. CT scan data are available on Morphosource under two different projects (details found in Data Availability Statement).
Randomization	None.
Blinding	None.

Did the study involve field work? ☐ Yes ☒ No

Reporting for specific materials, systems and methods

We require information from authors about some types of materials, experimental systems and methods used in many studies. Here, indicate whether each material, system or method listed is relevant to your study. If you are not sure if a list item applies to your research, read the appropriate section before selecting a response.

Materials & experimental systems

n/a	Involved in the study
☒	☐ Antibodies
☒	☐ Eukaryotic cell lines
☐	☒ Palaeontology and archaeology
☐	☒ Animals and other organisms
☒	☐ Clinical data
☒	☐ Dual use research of concern

Methods

n/a	Involved in the study
☒	☐ ChIP-seq
☒	☐ Flow cytometry
☒	☐ MRI-based neuroimaging

Palaeontology and Archaeology

Specimen provenance: Fossils of Yurlunggur are accessioned in a public institution (South Australian Museum, Adelaide, Australia). Portugalophis fossils are housed in the Museu Geologico, Lisbon, Portugal and are also publicly accessible. The extant snake and lizard samples are museum specimens from various publicly accessible collections (listed in the Supplementary Information and Supplementary Data 1). No new fossil material was collected for this study. No fossils were destructively sampled.

Specimen deposition: Fossils of Yurlunggur and Portugalophis are accessioned in publicly accessible institutions. CT scan data are available on Morphosource under two different projects: Teeth of Serpentes (https://www.morphosource.org/projects/000358042?locale=en); Modern and fossil snake tooth replacement (https://www.morphosource.org/projects/000395615?locale=en).

Dating methods: *If new dates are provided, describe how they were obtained (e.g. collection, storage, sample pretreatment and measurement), where they were obtained (i.e. lab name), the calibration program and the protocol for quality assurance OR state that no new dates are provided.*

☐ Tick this box to confirm that the raw and calibrated dates are available in the paper or in Supplementary Information.

Ethics oversight: Specimen access and permission to CT scan Portugalophis lignites (MG-LNEG 28094) was provided by Teresa Ponce Leao, Ruben Dias, Jose Moita, and Jorge Cerqueira (Museu Geologico, Lisbon). Permission to scan SAMA specimens was also acquired via the South Australian Research and Collections Committee.

Note that full information on the approval of the study protocol must also be provided in the manuscript.

Animals and other research organisms

Policy information about studies involving animals; ARRIVE guidelines recommended for reporting animal research, and Sex and Gender in Research

Laboratory animals: All extant animals were either osteological or fluid-preserved museum specimens. No animals were sacrificed for this study.

Wild animals: No wild animals were studied.

Reporting on sex: N/A

Field-collected samples: N/A

Ethics oversight: The corn snake sampled in this study was used in previous studies by A. S. Tucker and is housed in the Centre for Craniofacial Regenerative Biology (King's College London). The animal was cared for and killed at King's College London (KCL) according to UK Home Office license and regulations in line with those set out under the United Kingdom Animals (Scientific Procedures) Act 1986, the European Union Directive 2010/63/EU, and the Amendment Regulations 2012. Histological sampling of extant snakes from the South Australia Museum (SAMA) were approved by the South Australian Museum Research and Collections Committee.

Note that full information on the approval of the study protocol must also be provided in the manuscript.
